# Association Between Platelet Levels and 28-Day Mortality in Patients With Sepsis: A Retrospective Analysis of a Large Clinical Database MIMIC-IV

**DOI:** 10.3389/fmed.2022.833996

**Published:** 2022-04-07

**Authors:** Danni Wang, Suning Wang, Hao Wu, Jiansheng Gao, Kairong Huang, Danhong Xu, Huangyao Ru

**Affiliations:** Department of Critical Care, The First Affiliated Hospital of Guangdong Pharmaceutical University, Guangzhou, China

**Keywords:** intensive care unit, mortality, platelet count, sepsis, MIMIC-IV

## Abstract

**Background:**

This research focused on evaluating the correlation between platelet count and sepsis prognosis, and even the dose-response relationship, in a cohort of American adults.

**Method:**

Platelet counts were recorded retrospectively after hospitalization for patients admitted to Beth Israel Deaconess Medical Center’s intensive care unit between 2008 and 2019. On admission to the intensive care unit, sepsis patients were divided into four categories based on platelet counts (very low < 50 × 10^9^/L, intermediate-low 50 × 10^9^–100 × 10^9^/L, low 100 × 10^9^–150 × 10^9^/L, and normal ≥ 150 × 10^9^/L). A multivariate Cox proportional risk model was used to calculate the 28-day risk of mortality in sepsis based on baseline platelet counts, and a two-piece linear regression model was used to calculate the threshold effect.

**Results:**

The risk of 28-day septic mortality was nearly 2-fold higher in the platelet very low group when compared to the low group (hazard ratios [HRs], 2.24; 95% confidence interval [CI], 1.92–2.6). Further analysis revealed a curvilinear association between platelets and the sepsis risk of death, with a saturation effect predicted at 100 × 10^9^/L. When platelet counts were below 100 × 10^9^/L, the risk of sepsis 28-day death decreased significantly with increasing platelet count levels (HR, 0.875; 95% CI, 0.84–0.90).

**Conclusion:**

When platelet count was less than 100 × 10^9^/L, it was a strong predictor of the potential risk of sepsis death, which is declined by 13% for every 10 × 10^9^/L growth in platelets. When platelet counts reach up to 100 × 10^9^/L, the probability of dying to sepsis within 28 days climbs by 1% for every 10 × 10^9^/L increase in platelet count.

## Introduction

Low platelet counts are common in critically ill patients, both on admission to and during intensive care unit (ICU). The incidence of thrombocytopenia in ICU patients varies from 15 to 58%, depending on the value used to define thrombocytopenia (1, 2).

Thrombocytopenia is defined as a platelet count below 150 × 10^9^/L [BCSH (3)] and severe thrombocytopenia is defined as a platelet count below 50 × 10^9^/L. In a recent study, Yang et al. reported (4) that thrombocytopenia is common in patients with Coronavirus Disease-19 (COVID-19) and is associated with an increased risk of in-hospital mortality, with lower platelet counts associated with higher mortality. A study of 931 patients with sepsis collected from two ICUs in the Netherlands found that in-hospital thrombocytopenia was associated with increased mortality and a more disturbing host response during sepsis, independent of disease severity (5).

Sepsis is a life-threatening organ dysfunction that is caused by a dysregulated host response to infection (6). Sepsis and septic shocks are major medical problems that affect millions of people worldwide each year, with one-third to one-sixth of them dying from sepsis (7–9). Researchers have understood sepsis as a complex interplay of cytokine storm, systemic inflammation, endothelial dysfunction, capillary leakage, and pathological hemostasis (10–13). Microvascular thrombosis, microvascular occlusion, and hypoperfusion are the main causes of organ dysfunction during sepsis (14). In overwhelming sepsis, platelets contribute to activating the procoagulant cascade and subsequent complications associated with microvascular thrombosis and subsequent organ dysfunction (15). Platelets are the cornerstone of this process, and, therefore, thrombocytopenia may be a prognostic factor in infectious shock. Early detection and appropriate treatment within the first few hours after the onset of sepsis may improve the prognosis. The lowest point of platelet count that is assessed during the clinical course may represent an early warning to the clinician. Platelet counts are also readily available in routine practice. This simple bedside test can help in identifying critically ill patients without the need to calculate more complex and time-consuming scores.

There are conflicting data in the literature regarding the impact of thrombocytopenia on prognosis. To date, few studies have investigated the association between early platelet counts and mortality among the many identified risk factors for mortality (16). In addition, these studies have mainly considered ICU or in-hospital mortality (17, 18). Some authors (19–21) reported thrombocytopenia as an independent prognostic factor for ICU or hospital discharge mortality. In contrast, others did not find any such association or only found an effect depending on the instability of the patient’s clinical status (22). In addition, these existing studies suffer from several limitations, such as small sample sizes, inconsistent definitions of platelet count subgroups, observed endpoints, and adjustment for some important covariates.

The dose-response relationship between baseline platelet count levels and the risk of sepsis death has not been elucidated. A prospective study revealed a persistent negative association between platelet count and sepsis 28-day death without a threshold effect analysis (22). Therefore, this study is aimed to examine the association between baseline platelet counts and the risk of sepsis 28-day death and describe in detail the nature of the dose-response relationship.

## Methods

### Data Source

Data from the Marketplace for Medical Information in Intensive Care (MIMIC) database were used to conduct this study. Patients admitted to the ICU of Beth Israel Deaconess Medical Center from 2008 to 2019 were included in Johnson et al. (23). The raw data were extracted using Navicat using Structured Query Language (SQL) and further processed using R software. The MIMIC-IV database is publicly available from https://mimic.physionet.org/. The database is freely accessible, and any researcher who has accepted the data use agreement and completed the “Protection of Human Subjects” training can request to access to the data (24). We did not require patient consent or ethical approval, data information was de-identified, and patient identifiers were removed. Individuals who have completed the Collaborative Training Program exam (author Wang’s certification number 27714078) can access the database. The Strengthening the Reporting of Observational Studies in Epidemiology (STROBE) guidelines were followed (25).

### Study Population

**FIGURE 1:**
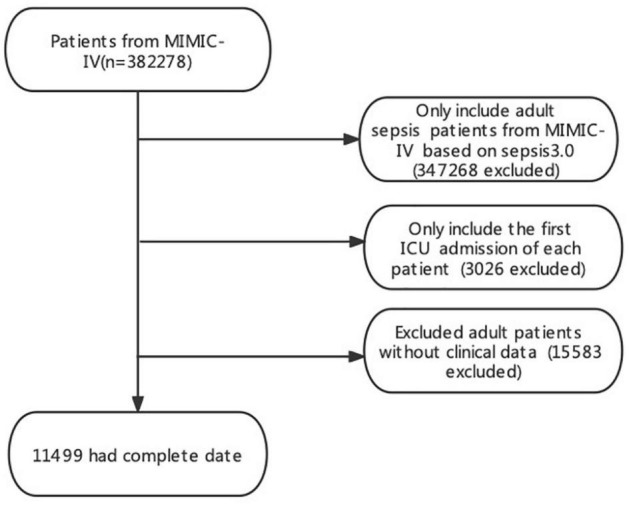
Study flowchart. MIMIC, Medical Information Mart for Intensive Care.

All patients in the database were selected. The inclusion criteria for this study were as follows: (1) sepsis was identified through the MIMIC-IV database and (2) adults (≥18 years) who were admitted to the ICU; exclusion criteria were as follows: (1) patients with the Sequential Organ Failure Assessment (SOFA) < 2; (2) patients with no vital signs or no platelets recorded were also excluded. The outcome cohort for the final analysis included 16,401 participants (Figure 1).

### Data Extraction and Management

We extracted platelet counts measured within 24 h of admission to the monitoring unit from MIMIC-IV for each included patient. The lowest platelet count was retained if multiple measurements were taken within the first 24 h. Platelet counts were then divided into four groups based on five groups of the SOFA scores: platelet counts below 50 × 10^9^/L, platelet counts between 50 × 10^9^/L and 100 × 10^9^/L, platelet counts between 100 × 10^9^/L and 150 × 10^9^/L, and platelet counts above 150 × 10^9^/L (26).

We also considered covariates that may affect the relationship between platelets and sepsis. We extracted the following basic data for each patient from MIMIC-IV: age, sex, race, body mass index (BMI), Elixhauser comorbid conditions (27), site of infection, organ support therapy, heart rate (HR), systolic blood pressure (SBP), diastolic blood pressure (DBP), and mean arterial pressure (MBP). The following biochemical tests were also collected for each patient: partial thromboplastin time (PTT), international normalized ratio (INR), prothrombin time (PT), white blood cell count (WBC), hemoglobin, blood urea nitrogen (BUN), and creatinine (Cr). Simplified acute physiology score (SAPS II) of patients was also recorded (28). The worst score and mean values of laboratory parameters and vital signs within 24 h of ICU admission were used in this study.

All scripts used for demographic characterization, severity score calculation, and comorbidity were obtained from the Github website (https://github.com/MIT-LCP/mimic-iv, access date. October 2021). Data extraction was performed with PostgreSQL tools (v9.6; PostgreSQL Global Development Group) using SQL.

### Primary and Secondary Results

The primary outcome was 28-day mortality. Baseline platelet count and 28-day risk of mortality from sepsis have a dose-response relationship. Secondary outcomes included length of stay in the ICU (LOS ICU) and LOS in the hospital (LOS hospital).

### Statistical Analysis

Data are expressed as mean ± standard deviation (SD) for continuous variables and frequencies or percentages for categorical variables. Statistical differences between platelet count subgroups were tested for baseline characteristic analysis with one-way ANOVA for continuous variables and chi-square tests for categorical variables. Cox proportional risk models were used to estimate the hazard ratio (HR) and 95% confidence interval (CI) for the association between platelet counts and 28-day mortality from sepsis. Unadjusted and multivariable-adjusted models were used. To assess confounding, we entered covariates into the Cox regression model in the base model or removed each covariate from the full model and compared regression coefficients. Covariates with initial regression coefficients that changed by more than 10% were included. In the multivariate model, we adjusted for age (years), sex (male or female), race (white or other), and BMI (<18.5, 18.5–25, 25–30, or ≥ 30.0 kg/m^2^) in model 1. In model 2, we further adjusted for congestive heart failure (CHF; yes or no), chronic obstructive pulmonary disease (COPD; yes or no), liver (yes or no), kidney (yes or no), diabetes (yes or no), metastatic tumors (yes or no), AIDS (yes or no), abdominal infections (yes or no), bloodstream infections (yes or no), respiratory infections (yes or no), and urinary tract infections (yes or no). In model 3, we further adjusted for catheter infection (yes or no), mechanical ventilation (MV; yes or no), renal replacement therapy (RRT; yes or no), SAPS II (≤40 or >40), ICU LOS, length of visit, hemoglobin, WBC, Cr, BUN, PT, INR, PTT, HR, SBP, DBP, and mean arterial pressure (MAP).

Trend tests were performed using linear regression by entering the median value of each subgroup of each platelet as a continuous variable in the model. A generalized additivity model was used to assess the non-linear relationship between platelets and sepsis prognosis. Based on the smoothed curves, we developed a two-segment linear regression model to identify threshold effects and adjust for potential confounders. Threshold levels of platelet counts were determined using a recursive approach that involved selecting turning points along with predefined intervals and selecting turning points that yielded a maximum likelihood model. The log-likelihood ratio test compared a two-segment linear regression model with a one-linear linear model. Using a stratified Cox regression model, subgroups were performed by age, gender, race, and severity scores. Interactions between subgroups were tested using likelihood ratio tests.

We used multiple imputation (MI), based on five replications and a chained equation approach method in the R MI procedure, to account for missing data. We performed several sensitivity analyses to assess the robustness of our findings. To ensure that our findings were not confounded by missing partial data, we further assessed whether indicator variables with missing data introduced bias in our results by performing multiple interpolation analyses. We also assessed the robustness of our main results by using a variety of analytical strategies, such as stratified analysis and multivariate Cox regression.

For all statistical analyses, we used the statistical packages R version 3.4.3 (R Foundation, Vienna, Austria). Two-sided values of *p* < 0.05 were considered statistically significant.

## Results

### Demographics and Baseline Characteristics

Of the 16,401 adult patients with sepsis (mean age 66.0 ± 15.6 years; 39.8% men), a total of 8,612 patients (52.5%) had no thrombocytopenia, whereas 47.5% (7,789/16,401) had thrombocytopenia [4,403 (26.8%) had platelet counts of 100 × 10^9^–150 × 10^9^/L; 2,463 (15%) had platelet counts of 50 × 10^9^–100 × 10^9^/L; and 923 (5.6%) had platelet counts < 50 × 10^9^/L]. The baseline characteristics of the study population according to platelet count are shown in Table 1. Participants with reduced platelet counts were more likely to be younger and more likely to have concurrent liver disorders, metastases, and AIDS; SAPS II scores averaged 40.9 ± 14.5, continuous renal replacement therapy accounted for 6.5%, and mechanical ventilation treatment accounted for 65.3%.

**TABLE 1:** Baseline and clinical characteristics of the study population according to platelet count.

	Total	Very low	Intermediate-low	Low	Normal	*P*-value
		(<50 × 10^9^/L)	(50 × 10^9^–100 × 10^9^/L)	(100 × 10^9^–150 × 10^9^/L)	(≥150 × 10^9^/L)	
Patients, n (%)	16,401	884 (5.4)	2,396 (14.6)	4,423 (27.0)	8,698 (53.0)	
**Demographics**						
Age (years)	66.0 ± 15.6	60.0 ± 14.8	65.3 ± 15.7	66.9 ± 14.9	66.3 ± 15.9	< 0.001
Male (%)	6,535 (39.8)	366 (41.4)	883 (36.9)	1,482 (33.5)	3,804 (43.7)	< 0.001
White (%)	11,118 (67.8)	582 (65.8)	1,582 (66)	3,054 (69)	5,900 (67.8)	0.043
BMI (kg/m^2^)	29.1 ± 7.4	28.2 ± 6.4	28.5 ± 6.5	28.9 ± 6.6	29.4 ± 8.1	< 0.001
**Chronic comorbidity, n (%)**						
CHF	5,267 (32.1)	179 (20.2)	620 (25.9)	1,390 (31.4)	3,078 (35.4)	< 0.001
COPD	4,473 (27.3)	197 (22.3)	566 (23.6)	1,081 (24.4)	2,629 (30.2)	< 0.001
Liver	2,521 (15.4)	447 (50.6)	785 (32.8)	552 (12.5)	737 (8.5)	< 0.001
Renal	3,890 (23.7)	183 (20.7)	561 (23.4)	1,048 (23.7)	2,098 (24.1)	0.148
Diabetes	4,079 (24.9)	189 (21.4)	493 (20.6)	1,084 (24.5)	2,313 (26.6)	< 0.001
Metastatic tumor	802 (4.9)	61 (6.9)	101 (4.2)	153 (3.5)	487 (5.6)	< 0.001
AIDS	121 (0.7)	20 (2.3)	23 (1)	17 (0.4)	61 (0.7)	< 0.001
Charlson	5.9 ± 2.9	6.4 ± 2.9	6.0 ± 2.9	5.7 ± 2.8	5.9 ± 2.9	< 0.001
**Primary source of infection, n (%)**						
Abdomen	49 (0.3)	6 (0.7)	9 (0.4)	11 (0.2)	23 (0.3)	0.157
Bloodstream	1,175 (7.2)	128 (14.5)	204 (8.5)	284 (6.4)	559 (6.4)	< 0.001
Catheter	43 (0.3)	2 (0.2)	7 (0.3)	8 (0.2)	26 (0.3)	0.657
Respiratory tract	1,576 (9.6)	97 (11)	225 (9.4)	364 (8.2)	890 (10.2)	0.001
Urinary tract	1,284 (7.8)	75 (8.5)	166 (6.9)	301 (6.8)	742 (8.5)	< 0.001
**Severity of disease**						
SAPS II	40.9 ± 14.5	48.2 ± 17.7	43.0 ± 14.9	39.7 ± 13.9	40.2 ± 14.1	< 0.001
**Organ support therapy, n (%)**						
RRT	1,070 (6.5)	127 (14.4)	204 (8.5)	232 (5.2)	507 (5.8)	< 0.001
MV	10,709 (65.3)	10,709 (65.3)	605 (68.4)	1,666 (69.5)	2,874 (65)	< 0.001
**Clinical data**						
Hemoglobin (g/dL)	9.7 ± 2.1	8.1 ± 2.0	8.9 ± 2.0	9.6 ± 2.0	10.0 ± 2.1	< 0.001
WBC (× 10^9^/L)	15.8 ± 11.5	14.4 ± 26.3	14.7 ± 12.4	14.9 ± 9.6	16.7 ± 9.4	< 0.001
Cr (mg/dL)	1.7 ± 1.8	2.1 ± 1.7	1.9 ± 1.8	1.7 ± 2.0	1.7 ± 1.8	< 0.001
BUN (mg/dL)	31.8 ± 25.3	40.1 ± 28.4	33.1 ± 26.5	29.7 ± 24.0	31.6 ± 25.0	< 0.001
PT (s)	18.7 ± 13.1	24.3 ± 16.1	20.8 ± 14.1	18.1 ± 11.9	17.8 ± 12.8	< 0.001
INR	1.7 ± 1.3	2.3 ± 1.5	1.9 ± 1.3	1.7 ± 1.2	1.6 ± 1.3	< 0.001
PTT (s)	45.2 ± 30.4	54.6 ± 34.7	50.7 ± 32.4	44.1 ± 29.1	43.2 ± 29.6	< 0.001
HR (bpm)	87.0 ± 16.1	93.5 ± 17.7	87.3 ± 16.3	85.2 ± 15.2	87.1 ± 16.1	< 0.001
SBP (mmHg)	114.5 ± 14.7	111.8 ± 14.4	112.5 ± 14.1	113.7 ± 13.3	115.7 ± 15.4	< 0.001
DBP (mmHg)	60.8 ± 10.0	61.5 ± 10.0	60.1 ± 10.1	60.0 ± 9.6	61.4 ± 10.2	< 0.001
MAP (mmHg)	76.2 ± 9.8	75.3 ± 9.9	75.4 ± 9.9	75.7 ± 9.1	76.7 ± 10.1	< 0.001

*Variables are presented as mean ± SD or N (%). BMI, body mass index; CHF, congestive heart failure; COPD, chronic obstructive pulmonary disease; Charlson, Modified Charlson comorbidity index; SAPS II, Simplified Acute Physiology Score II; MV, mechanical ventilation; RRT, renal replacement therapy; WBC, white blood cell count; Cr, creatinine; BUN, blood urea nitrogen; PT, prothrombin time; INR, international normalized ratio; PTT, partial thromboplastin time; HR, heart rate; SBP, systolic blood pressure; DBP, diastolic blood pressure; MAP, mean arterial pressure.*

**FIGURE 2:**
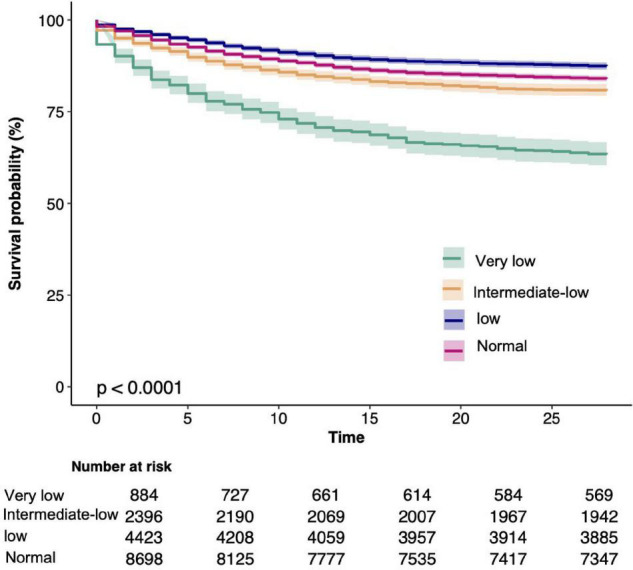
Kaplan-Meier Survival Curves for day 28 of sepsis patients depending on the platelet count.

Univariate analysis of clinical outcomes calculated mortality and hospital length and ICU LOS stratified by platelet count are summarized in Table 2. Participants with reduced platelet count had longer hospital and ICU LOS. In addition, 37.5% of patients with very low platelet count were died during hospitalization (*p* < 0.001); 35.9% of patients with very low platelet count were killed during 28 days of hospitalization (*p* < 0.001); and 43.3% of patients with very low platelet count were died within 1 year (*p* < 0.001). Kaplan-Meier curves for 28-day survival by platelet count are shown in Figure 2.

**TABLE 2:** Outcome of sepsis patients stratified according to platelet counts.

	Total	Very low	Intermediate-low	Low	Normal	*P*
		(<50 × 10^9^/L)	(50 × 10^9^–100 × 10^9^/L)	(100 × 10^9^–150 × 10^9^/L)	(≥150 × 10^9^/L)	
Patients (%)	16,401	884 (5.4)	2,396 (14.6)	4,423 (27.0)	8,698 (53.0)	
**Length of stay**						
Length of ICU stay (d)	6.3 ± 7.6	7.0 ± 7.7	6.0 ± 6.8	5.5 ± 7.0	6.8 ± 8.0	<0.001
Length of hospital stay (d)	13.5 ± 17.0	17.7 ± 19.4	14.2 ± 16.8	11.9 ± 11.9	13.7 ± 18.8	<0.001
**Mortality, n (%)**						
In-hospital mortality	2,814 (17.2)	338 (38.2)	486 (20.3)	568 (12.8)	1,422 (16.3)	<0.001
28-day mortality	2,736 (16.7)	324 (36.7)	462 (19.3)	557 (12.6)	1,393 (16)	<0.001
Acute kidney injury	12,858 (78.4)	735 (83.1)	1,902 (79.4)	3,378 (76.4)	6,843 (78.7)	<0.001

*Notes: Variables are presented as mean ± SD or N (%).*

### Association Between Platelets and Prognosis of Sepsis

In our study, patients with sepsis recorded 2,736 (16.7) deaths at 28 days, 2,984 (18.2) deaths at 60 days, 3,086 (18.8) deaths at 90 days, and 3,387 (20.7) deaths at 1 year. In the unadjusted model, an increased risk of 28-day death occurred with a decrease in platelet count when compared with participants with 100 × 10^9^–150 × 10^9^/L (*p* < 0.066 for trend). Participants with platelet counts at ≤50 × 10^9^/L vs. 100 × 10^9^ to 150 × 10^9^/L had a 3-fold 3.31 (2.89–3.79) increase in the odds of developing 28-day death. We also found that as platelet counts were increased, the odds of 28-day mortality were increased 1-fold 1.28 (1.16–1.42) for participants with platelet counts ≥ 150 × 10^9^/L vs. 100 × 10^9^–150 × 10^9^/L. After multivariate adjustment, such as age, sex, race, BMI, chronic disease, source of infection, interventions, severity score, and presence of laboratory findings, lower or higher platelet counts were significantly associated with lower long-term and short-term survivals in patients with sepsis (Table 3). The multivariable-adjusted HR and 95% CI from the platelet count categories (<50 × 10^9^/L, 50 × 10^9^–100 × 10^9^/L, 100 × 10^9^–150 × 10^9^/L, ≥150 × 10^9^/L) were 2.31 (1.99–2.68), 1.34 (1.18–1.51), 1.00 (reference), and 1.17 (1.06–1.29) for 28-day mortality (*p*_*trend*_ = 0.262); 2.28 (1.98–2.63), 1.32 (1.17–1.49), 1.00 (reference), and 1.17 (1.06–1.28) for 60-day mortality, respectively (*p*_*trend*_ = 0.239); and 2.27 for 90-day mortality (1.97–2.6), 1.33 (1.18–1.49), 1.00 (reference), and 1.18 (1.08–1.3), respectively, (*p*_*trend*_ = 0.125); and 1-year mortality rates of 2.3 (2.01–2.62), 1.32 (1.18–1.48), 1.00 (reference), and 1.19 (1.09–1.3), respectively (*p*_*trend*_ = 0.085).

**TABLE 3:** Relationship between platelet count and 28-day mortality in patients with sepsis.

	Low	Very low	Intermediate-low	Normal	P_*trend*_
	(100 × 10^9^–150 × 10^9^/L)	(<50 × 10^9^/L)	(50 × 10^9^–100 × 10^9^/L)	(≥150 × 10^9^/L)	
**28-day mortality**					
Number of deaths/total	557/4,423	324/884	462/2,396	1,393/8,698	
Crude Model	1.0	3.43 (2.99–3.93)	1.6 (1.42–1.81)	1.3 (1.18–1.43)	0.042
Model 1	1.0	3.79 (3.3–4.35)	1.61 (1.43–1.83)	1.28 (1.16–1.41)	0.132
Model 2	1.0	2.72 (2.35–3.14)	1.39 (1.22–1.57)	1.24 (1.12–1.37)	0.07
Model 3	1.0	2.24 (1.92–2.6)	1.35 (1.19–1.53)	1.21 (1.1–1.34)	0.041

*Data presented are HRs and 95% CIs.*

*Model 1: adjusted for age, Sex, Ethnicity, and BMI;*

*Model 2: further adjusted (from Model 1) for CHF, COPD, Liver, Renal, Diabetes, Metastatic tumor, AIDS, Charlson, Abdomen, Bloodstream, Catheter, Respiratory tract, Urinary tract;*

*Model 3: further adjusted (from Model 2) for MV, RRT, SAPS II, Length of ICU stay, Length of hospital stay, Hemoglobin, WBC, Cr, BUN, PT, INR, PTT, HR, SBP, DBP, MAP. BMI, body mass index; CHF, congestive heart failure; COPD, chronic obstructive pulmonary disease; SAPS II, Simplified Acute Physiology Score II; MV, mechanical ventilation; RRT, renal replacement therapy; WBC, white blood cell count; Cr, creatinine; BUN, blood urea nitrogen; PT, prothrombin time; INR, international normalized ratio; PTT, partial thromboplastin time; HR, heart rate; SBP, systolic blood pressure; DBP, diastolic blood pressure; MAP, mean arterial pressure.*

### Threshold Effect Analysis of Platelet Count on Sepsis Mortality

**FIGURE 3:**
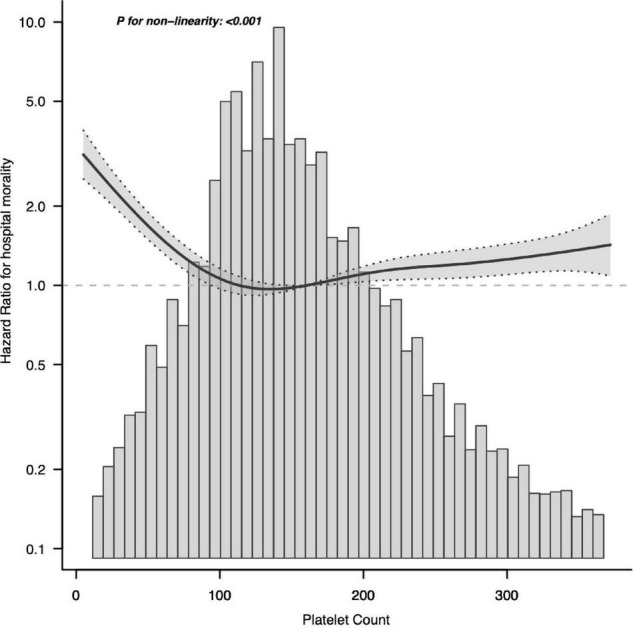
Associations between platelet count concentrations with 28-day mortality among participants with sepsis in MIMIC-IV. HRs were adjusted for age, sex, ethnicity, BMI, CHF, COPD, liver, renal, diabetes, metastatic tumor, AIDS, Charlson, abdomen, bloodstream, respiratory tract, urinary tract, catheter, MV, RRT, SAPS II, length of ICU stay, length of hospital stay, hemoglobin, WBC, Cr, BUN, PT, INR, PTT, HR, SBP, DBP, and MAP. Both *p* linearity, 0.001. BMI, body mass index; CHF, congestive heart failure; COPD, chronic obstructive pulmonary disease; SAPS II, Simplified Acute Physiology Score II; MV, mechanical ventilation; RRT, renal replacement therapy; WBC, white blood cell count; Cr, creatinine; BUN, blood urea nitrogen; PT, prothrombin time; INR, international normalized ratio; PTT, partial thromboplastin time; HR, heart rate; SBP, systolic blood pressure; DBP, diastolic blood pressure; MAP, mean arterial pressure.

We used smoothing function analysis to assess whether there is a dose-response relationship between platelets and 28-day mortality events in sepsis. After adjusting for potential confounders, a non-linear relationship was observed between serum platelet counts and sepsis 28-day mortality events. The results also applied to the long-term prognostic outcomes of septic patients (Figure 3). Further analysis revealed a curvilinear association between platelets and the occurrence of sepsis 28-day mortality events, with a saturation effect predicted at 100 × 10^9^/L. The risk of developing sepsis 28-day mortality was significantly decreased with increasing platelet count levels when platelet counts were below 100 × 10^9^/L (HR 0.875; 95% CI, 0.847–0.903). When platelet counts exceeded 100 × 10^9^/L, the risk of sepsis 28-day death was increased by 1% for each 10 × 10^9^/L increase in platelet counts (Table 4).

**TABLE 4:** Threshold effect analysis for the relationship between Platelet Count and 28-day mortality.

Outcome:	HR (95% CI)	*P*-value
One-line linear regression model	1.0 (1.0, 1.0)	0.001
**Two-piecewise linear regression model**		
<100	0.875 (0.847, 0.903)	<0.001
≥100	1.014 (1.007, 1.021)	<0.001
Log-likelihood ratio test	<0.001	

*HRs were adjusted for Age, Sex, Ethnicity, and BMI, CHF, COPD, Liver, Renal, Diabetes, Metastatic tumor, AIDS, Charlson, Abdomen, Bloodstream, Respiratory tract, Urinary tract, Catheter, MV, RRT, SAPS II, Length of ICU stay, Length of hospital stay, Hemoglobin, WBC, Cr, BUN, PT, INR, PTT, HR, SBP, DBP, MAP.*

In the graph, the black line indicates the estimated risk of septic death, and the gray band indicates the point-by-point 95% CI adjusted for age, sex, laboratory results, etc.

### Subgroup Analyses

We performed stratified and interaction analyses to see if the association between platelet count and sepsis 28-day mortality was stable in different subgroups. Consistent results were observed when the analysis was stratified by age, sex, race, BMI, associated comorbidities, source of infection, severity score, and intervention treatment (Supplementary Table 2). The data showed an interaction between SAPA II in the association between platelet count and 28-day mortality events in sepsis (interaction *p* = 0.004). For participants with SAPS II < 56, the risk of mortality increased with decreasing platelet levels, [<50 × 10^9^/L 2.57 (2.11–3.13); 50 × 10^9^ to 100 × 10^9^/L 1.38 (1.17–1.63); and >150 × 10^9^/L 1.24 (1.09–1.4) vs. 100 × 10^9^ to 150 × 10^9^/L 1.00, *p* for trend = 0.004]. Similarly, significant interactions were detected between platelet count and the presence of comorbid liver disorders, metastases, respiratory tract infections, urinary tract infections, and clinical interventions for treatment stratified variables (all *p*_*trend*_ < 0.05). There were no significant associations among those with or without comorbidity (chronic obstructive pulmonary, renal, and diabetes). Although the differences were not statistically significant (*p* > 0.05 for interaction), similar results were found across gender, race, and bloodstream infection status.

### Sensitivity Analysis

We performed several sensitivity analyses to test the robustness of our findings. First, considering the presence of missing partial laboratory data, we further performed multiple interpolation of the missing data and performed multiple regression analysis that did not significantly alter our findings (Supplementary Table 4). Secondly, considering the possibility of organ impairment in patients, we assessed the presence of acute kidney injury and concomitant disseminated intravascular coagulation (DIC) in patients, which could still explain our findings (Supplementary Tables 5, 6).

## Discussion

This large retrospective cohort study of the US adults with sepsis found that platelet count was significantly associated with a 28-day risk of death from sepsis. The association was independent of traditional risk factors, such as sex, race, BMI, comorbidities, and source of infection. Various sensitivity analyses and stratified analyses demonstrated the robustness of these findings. Our findings confirm a non-linear association between platelet count and 28-day risk of death. This relationship was characterized as follows: when platelet counts were below 100 × 10^9^/L, the risk of 28-day death from sepsis was reduced by 13% for every 10 × 10^9^/L increase in platelet count. When platelet counts were above 100 × 10^9^/L, the risk of sepsis death at 28 days was increased by 1% for every 10 × 10^9^/L increase in platelet count. A platelet count that is sensitive to change may help strengthen the therapist’s ability to assess prognosis a few days after a patient is admitted to the ICU, thereby improving treatment decisions.

In addition to their important role in hemostasis, platelets play an important role in inflammatory diseases. Studies have shown that platelets are also cells that have immunogenic capabilities. Like traditional congenital immune cells, platelets are immediately recruited into injured and inflamed tissues, they release immune media, express and fall off to immunoactive membrane receptors, which interact with other immunocytes, identify and remove pathogens (29). Sepsis is caused by a dysregulated host response to infection and can lead to organ dysfunction, permanent disability, or death. During sepsis, tissue damage results while uncontrolled complement, coagulation, inflammatory systems, and platelet dysfunction. The balance between systemic inflammatory response syndrome and compensatory anti-inflammatory response (CARS) regulates the outcome of sepsis. Persistent low platelet count is considered an independent risk factor for death in sepsis. In general, 20–58% of patients with sepsis develop a low platelet count, of which 10% develop a severely low platelet count (30). Variations in reported values may arise from patient heterogeneity, different inclusion criteria, pathogens, and other factors. In sepsis, low platelet counts may be regulated by altered platelet production or phagocytosis or by platelet clearance in the circulation due to platelet-leukocyte or platelet-pathogen interactions, vascular injury, or deoxygenation (31). Some studies have shown that thrombocytopenia is associated with the prognosis of sepsis death (5, 22). Our findings on platelet counts are consistent with these studies. They found that thrombocytopenia is an important predictor of short-term death in sepsis. However, what degree of platelet count depression has not been clearly stated. The current study did not describe its dose-response relationship in detail. Our research found that patients with thrombocytopenic infectious shock were more severely ill, had higher SAPS II scores, and required more organ function support and hospital stay than non-thrombocytopenic patients. Moreover, advanced age, men, cirrhosis, respiratory, urinary tract, and bloodstream infections were risk factors for thrombocytopenia. These findings are consistent with previous studies (16, 21, 32, 33).

Strengths of the current study include the relatively large sample size provided by MIMIC-IV and the use of a representative sample of United States adults with sepsis, which facilitates the generalization of our findings. Most previous studies on the prognostic impact of thrombocytopenia, the dose-response relationship between baseline platelet count levels and the risk of death from sepsis has not been elucidated. The aim of this study was to investigate the relationship between baseline platelet counts and the risk of death from sepsis at 28 days and to describe in detail the nature of the dose-response relationship. In addition, given the comprehensive data obtained in MIMIC-IV, we adjusted for a variety of potential confounders, such as race/ethnicity, BMI, comorbidity, source of infection, severity score, and level of laboratory indicators. However, our study also had the limitations of most retrospective studies; first, causality could not be determined due to the observational study design. Second, it was not designed to determine the cause of platelet count decline in ICU patients; therefore, we could not speculate on the pathophysiological mechanism of increased mortality in patients with decreased platelet counts. The most common cause of postoperative thrombocytopenia (33, 34) or bluntly increased platelet counts has been reported to be sepsis-related disseminated intravascular coagulation, which is more common than liver disease, blood disorders, massive transfusions, pharmacologic thrombocytopenia, and immune-mediated thrombocytopenia. Therefore, a decreased platelet count may be a powerful marker for assessing patient prognosis regardless of the mechanism. This study should serve as the basis for future well-designed studies to evaluate the impact of decreased platelet counts on mortality and causality.

## Conclusion

A representative sample of the United States adults with sepsis found that lower platelet counts were significantly associated with higher 28-day mortality. These findings support the potential benefit of maintaining a normal platelet count status in the sepsis clinic in preventing premature death in septic patients.

## Data Availability Statement

The raw data supporting the conclusions of this article will be made available by the authors, without undue reservation.

## Ethics Statement

Ethical review and approval was not required for the study on human participants in accordance with the local legislation and institutional requirements. Written informed consent for participation was not required for this study in accordance with the national legislation and the institutional requirements.

## Author Contributions

DW conducted data analysis and wrote the manuscript. SW conducted data analysis and modified the manuscript. HW and JG conducted the data collection. KH conducted the data collection and data interpretation. DX drew the figure. HR study manuscript design, data collection and analysis, manuscript preparation, and review. All authors read and approved the final manuscript.

## Conflict of Interest

The authors declare that the research was conducted in the absence of any commercial or financial relationships that could be construed as a potential conflict of interest.

## Publisher’s Note

All claims expressed in this article are solely those of the authors and do not necessarily represent those of their affiliated organizations, or those of the publisher, the editors and the reviewers. Any product that may be evaluated in this article, or claim that may be made by its manufacturer, is not guaranteed or endorsed by the publisher.

## Abbreviations

BMI: body mass index
CHF: congestive heart failure
COPD: chronic obstructive pulmonary disease
Charlson: Modified Charlson comorbidity index
SAPS II: Simplified Acute Physiology Score II
MV: mechanical ventilation
RRT: renal replacement therapy
WBC: white blood cell count
Cr: creatinine
BUN: blood urea nitrogen
PT: prothrombin time
INR: international normalized ratio
PTT: partial thromboplastin time
HR: heart rate
SBP: systolic blood pressure
DBP: diastolic blood pressure
MAP: mean arterial pressure.
