# Supplementary material for: Association Between Platelet Levels and 28-Day Mortality in Patients With Sepsis: A Retrospective Analysis of a Large Clinical Database MIMIC-IV
Source: Front Med (Lausanne). 2022 Apr 7;9:833996. doi: 10.3389/fmed.2022.833996 (PMC9021789; doi:10.3389/fmed.2022.833996)
Supplement: Supplementary file 1 [file Data_Sheet_1.docx]

**Appendix**

**Supplementary Table 1.** Univariate analysis for 28-day mortality

| Covariate | HR(95%CI) | P |
| --- | --- | --- |
| **Age (years)** |  | < 0.001 |
| ＜65 | 1.0 |  |
| ≥65 | 1.53 (1.41,1.65) | < 0.001 |
| **Gender** |  | < 0.001 |
| female | 1.0 |  |
| male | 1.14 (1.06,1.23) | < 0.001 |
| **Race** |  | < 0.001 |
| other | 1.0 |  |
| white | 0.72 (0.67,0.78) | < 0.001 |
| **BMI ( kg/m2)** |  | < 0.001 |
| 18.5-25 | 1.0 |  |
| ＜18.5 | 1.33 (1.1,1.6) | 0.003 |
| 25-30 | 0.82 (0.74,0.9) | < 0.001 |
| ≥30 | 0.75 (0.68,0.82) | < 0.001 |
| **Chronic comorbidity** |  |  |
| CHF | 1.48 (1.37,1.6) | < 0.001 |
| COPD | 1.2 (1.1,1.3) | < 0.001 |
| Mliver | 1.89 (1.73,2.06) | < 0.001 |
| Renal | 1.5 (1.39,1.63) | < 0.001 |
| Diabetes | 0.96 (0.88,1.04) | 0.321 |
| Metastatic tumor | 2.56 (2.27,2.9) | < 0.001 |
| AIDS | 1.0086 (0.6497,1.5659) | 0.969 |
| Charlson | 1.17 (1.16,1.19) | < 0.001 |
| **Primary source of infection** |  |  |
| Abdomen | 1.06 (0.55,2.04) | 0.859 |
| Bloodstream | 1.65 (1.46,1.86) | < 0.001 |
| Catheter | 1.21 (0.63,2.33) | 0.563 |
| Respiratory tract | 1.55 (1.39,1.72) | < 0.001 |
| Urinary tract | 1.46 (1.3,1.65) | < 0.001 |
| **Severity of disease** |  |  |
| SAPS II |  | < 0.001 |
| SAPS II ＜56 | 1.0 |  |
| SAPS II ≥56 | 4.1 (3.79,4.43) | < 0.001 |
| **Organ support therapy** |  |  |
| RRT | 2.41 (2.15,2.69) | < 0.001 |
| MV | 2.34 (2.13,2.57) | < 0.001 |
| **Clinical data** |  |  |
| Hemoglobin (g/dL) | 0.96 (0.94,0.97) | < 0.001 |
| WBC (× 10^9^/L) | 1.0084 (1.0068,1.0101) | < 0.001 |
| Cr (mg/dL) | 1.05 (1.05,1.06) | < 0.001 |
| BUN (mg/dL) | 1.01 (1.01,1.01) | < 0.001 |
| PT (s) | 1.02 (1.01,1.02) | < 0.001 |
| INR | 1.15 (1.14,1.17) | < 0.001 |
| PTT (s) | 1.0091 (1.0081,1.01) | < 0.001 |
| HR (bpm) | 1.02 (1.02,1.02) | < 0.001 |
| SBP (mmHg) | 0.98 (0.98,0.98) | < 0.001 |
| DBP (mmHg) | 0.991 (0.9871,0.9948) | < 0.001 |
| MAP (mmHg) | 0.98 (0.98,0.98) | < 0.001 |
| **Length of stay** |  |  |
| Length of ICU stay (d) | 1.0067 (1.0025,1.0109) | 0.002 |
| Length of hospital stay (d) | 0.96 (0.95,0.96) | < 0.001 |

Notes: data presented are HRs and 95% CIs.

Abbreviations: BMI, body mass index; CHF, congestive heart failure; COPD, chronic obstructive pulmonary disease; Charlson, Modified Charlson comorbidity index; SAPS II, Simplified Acute Physiology Score II; MV, mechanical ventilation; RRT, renal replacement therapy; WBC, white blood cell count; Cr, creatinine; BUN, blood urea nitrogen; PT, prothrombin time; INR, international normalized ratio; PTT, partial thromboplastin time; HR, heart rate; SBP, systolic blood pressure; DBP, diastolic blood pressure; MAP, mean arterial pressure.

**Supplementary Table 2.** Stratified analyses of the associations (hazard ratios, 95% CIs) between Platelet Count and 28-day mortality.

| **Subgroup** | Low | Very low | Intermediate-low | Normal | P _trend_ | P_interaction_ |
| --- | --- | --- | --- | --- | --- | --- |
|  | (100x 10^9^ to 150 x 10^9^/L) | (<50x10^9^/L) | (50x10^9^ to100 x 10^9^/L) | (≥150 x 10^9^/L) |  |  |
| **Age (years)** |  |  |  |  |  | 0,001 |
| ＜65 | 1.0 | 2.07 (1.63~2.63) | 1.65 (1.33~2.04) | 1.2 (0.99~1.44) | 0.266 |  |
| ≥ 65 | 1.0 | 2.37 (1.94~2.9) | 1.19 (1.01~1.4) | 1.23 (1.1~1.39) | 0.036 |  |
| **Gender** |  |  |  |  |  | 0.266 |
| female | 1.0 | 2.28 (1.88~2.76) | 1.24 (1.05~1.46) | 1.18 (1.04~1.35) | 0.247 |  |
| male | 1.0 | 2.25 (1.77~2.86) | 1.5 (1.23~1.84) | 1.27 (1.08~1.49) | 0.065 |  |
| **Race** |  |  |  |  |  | 0.387 |
| other | 1.0 | 2.13 (1.67~2.71) | 1.42 (1.16~1.73) | 1.11 (0.95~1.31) | 0.753 |  |
| white | 1.0 | 2.29 (1.89~2.79) | 1.29 (1.09~1.52) | 1.25 (1.1~1.42) | 0.054 |  |
| **BMI ( kg/m2)** |  |  |  |  |  | 0.001 |
| 18.5-25 | 1.0 | 2.46 (1.88~3.22) | 0.99 (0.77~1.26) | 1.06 (0.89~1.27) | 0.599 |  |
| ≤18.5 | 1.0 | 1.06 (0.35~3.25) | 1.37 (0.72~2.6) | 0.96 (0.55~1.68) | 0.802 |  |
| 25-30 | 1.0 | 2.02 (1.53~2.67) | 1.28 (1.01~1.61) | 1.25 (1.04~1.5) | 0.126 |  |
| ≥30 | 1.0 | 2.55 (1.97~3.31) | 1.76 (1.42~2.18) | 1.33 (1.12~1.58) | 0.027 |  |
| **CHF** |  |  |  |  |  | 0.002 |
| No | 1.0 | 2.07 (1.71~2.5) | 1.47 (1.25~1.73) | 1.35 (1.17~1.55) | 0.003 |  |
| Yes | 1.0 | 2.65 (2.06~3.42) | 1.12 (0.91~1.38) | 1.04 (0.9~1.2) | 0.552 |  |
| **COPD** |  |  |  |  |  | 0.157 |
| No | 1.0 | 2.27 (1.9~2.72) | 1.42 (1.22~1.65) | 1.29 (1.14~1.46) | 0.01 |  |
| Yes | 1.0 | 2.29 (1.72~3.05) | 1.19 (0.94~1.51) | 1.07 (0.9~1.28) | 0.824 |  |
| **Mliver** |  |  |  |  |  | <0,001 |
| No | 1.0 | 3.26 (2.7~3.93) | 1.32 (1.13~1.54) | 1.24 (1.11~1.39) | 0.063 |  |
| Yes | 1.0 | 1.39 (1.08~1.8) | 1.18 (0.94~1.49) | 1.12 (0.88~1.41) | 0.674 |  |
| **Renal** |  |  |  |  |  | 0.167 |
| No | 1.0 | 2.17 (1.8~2.61) | 1.37 (1.17~1.6) | 1.24 (1.09~1.4) | 0.048 |  |
| Yes | 1.0 | 2.15 (1.63~2.84) | 1.19 (0.95~1.5) | 1.16 (0.98~1.38) | 0.383 |  |
| **Diabetes** |  |  |  |  |  | 0.664 |
| No | 1.0 | 2.2 (1.85~2.61) | 1.37 (1.18~1.58) | 1.27 (1.13~1.42) | 0.011 |  |
| Yes | 1.0 | 2.48 (1.81~3.4) | 1.26 (0.96~1.66) | 1.11 (0.91~1.35) | 0.999 |  |
| **Metastatic tumor** |  |  |  |  |  | 0.036 |
| No | 1.0 | 2.18 (1.86~2.56) | 1.4 (1.23~1.6) | 1.23 (1.11~1.37) | 0.019 |  |
| Yes | 1.0 | 1.87 (1.18~2.97) | 0.66 (0.41~1.05) | 0.87 (0.63~1.2) | 0.093 |  |
| **Bloodstream** |  |  |  |  |  | 0.113 |
| No | 1.0 | 2.27 (1.92~2.67) | 1.33 (1.16~1.52) | 1.24 (1.12~1.38) | 0.018 |  |
| Yes | 1.0 | 2.11 (1.39~3.22) | 1.4 (0.96~2.03) | 0.89 (0.65~1.21) | 0.119 |  |
| **Respiratory tract** |  |  |  |  |  | 0.005 |
| No | 1.0 | 2.3 (1.95~2.7) | 1.39 (1.22~1.59) | 1.24 (1.11~1.38) | 0.026 |  |
| Yes | 1.0 | 1.65 (1.08~2.5) | 1.04 (0.73~1.49) | 0.91 (0.7~1.19) | 0.233 |  |
| **Urinary tract** |  |  |  |  |  | 0.036 |
| No | 1.0 | 2.35 (2.01~2.76) | 1.41 (1.23~1.61) | 1.26 (1.14~1.4) | 0.01 |  |
| Yes | 1.0 | 1.58 (0.99~2.52) | 0.86 (0.57~1.3) | 0.83 (0.61~1.12) | 0.085 |  |
| **Severity of disease** |  |  |  |  |  | 0.004 |
| SAPS II＜56 | 1.0 | 2.57 (2.11~3.13) | 1.38 (1.17~1.63) | 1.24 (1.09~1.4) | 0.043 |  |
| SAPS II ≥ 56 | 1.0 | 1.81 (1.44~2.29) | 1.19 (0.97~1.46) | 1.08 (0.91~1.28) | 0.847 |  |
| **RRT** |  |  |  |  |  | <0,001 |
| No | 1.0 | 2.6 (2.21~3.06) | 1.29 (1.13~1.48) | 1.2 (1.08~1.34) | 0.118 |  |
| Yes | 1.0 | 1.07 (0.71~1.62) | 1.31 (0.92~1.88) | 1.23 (0.9~1.67) | 0.139 |  |
| **MV** |  |  |  |  |  | <0,001 |
| No | 1.0 | 2.83 (2.05~3.93) | 1.1 (0.81~1.48) | 0.95 (0.76~1.19) | 0.082 |  |
| Yes | 1.0 | 2.07 (1.75~2.46) | 1.35 (1.17~1.55) | 1.24 (1.11~1.39) | 0.016 |  |

Abbreviations: BMI, body mass index; CHF, congestive heart failure; COPD, chronic obstructive pulmonary disease; Charlson, Modified Charlson comorbidity index; SAPS II, Simplified Acute Physiology Score II; MV, mechanical ventilation; RRT, renal replacement therapy; WBC, white blood cell count; Cr, creatinine; BUN, blood urea nitrogen; PT, prothrombin time; INR, international normalized ratio; PTT, partial thromboplastin time; HR, heart rate; SBP, systolic blood pressure; DBP, diastolic blood pressure; MAP, mean arterial pressure.

**Supplementary Table 3.** Relationship between platelet count and 28-day mortality in patients with sepsis.

| Covariate | crude.HR_95CI | crude.P_value | adj.HR_95CI | adj.P_value |
| --- | --- | --- | --- | --- |
| **Age (years)** |  |  |  |  |
| ≥65 | 1.53 (1.41,1.65) | < 0.001 | 1.12 (1.01~1.24) | 0.029 |
| **Gender** |  |  |  |  |
| male | 1.14 (1.06,1.23) | < 0.001 | 1.15 (1.07~1.25) | <0.001 |
| **Race** |  |  |  |  |
| white | 0.72 (0.67,0.78) | < 0.001 | 0.78 (0.72~0.84) | <0.001 |
| **BMI ( kg/m2)** |  |  |  |  |
| 18.5-25 | 1.0 |  | 1.0 |  |
| ＜18.5 | 1.33 (1.1,1.6) | 0.003 | 1.15 (0.96~1.39) | 0.136 |
| 25-30 | 0.82 (0.74,0.9) | < 0.001 | 0.83 (0.75~0.91) | <0.001 |
| ≥30 | 0.75 (0.68,0.82) | < 0.001 | 0.78 (0.71~0.86) | <0.001 |
| **Chronic comorbidity** |  |  |  |  |
| CHF | 1.48 (1.37,1.6) | < 0.001 | 0.96 (0.87~1.05) | 0.377 |
| COPD | 1.2 (1.1,1.3) | < 0.001 | 0.88 (0.81~0.96) | 0.004 |
| Mliver | 1.89 (1.73,2.06) | < 0.001 | 1.36 (1.22~1.51) | <0.001 |
| Renal | 1.5 (1.39,1.63) | < 0.001 | 0.77 (0.69~0.87) | <0.001 |
| Diabetes | 0.96 (0.88,1.04) | 0.321 | 0.8 (0.73~0.88) | <0.001 |
| Metastatic tumor | 2.56 (2.27,2.9) | < 0.001 | 1.02 (0.84~1.23) | 0.863 |
| AIDS | 1.0086 (0.6497,1.5659) | 0.969 | 0.34 (0.21~0.54) | <0.001 |
| Charlson | 1.17 (1.16,1.19) | < 0.001 | 1.17 (1.14~1.2) | <0.001 |
| **Primary source of infection** |  |  |  |  |
| Abdomen | 1.06 (0.55,2.04) | 0.859 | 0.89 (0.46~1.73) | 0.74 |
| Bloodstream | 1.65 (1.46,1.86) | < 0.001 | 1.29 (1.13~1.46) | <0.001 |
| Catheter | 1.21 (0.63,2.33) | 0.563 | 0.78 (0.4~1.52) | 0.473 |
| Respiratory tract | 1.55 (1.39,1.72) | < 0.001 | 1.22 (1.08~1.38) | 0.001 |
| Urinary tract | 1.46 (1.3,1.65) | < 0.001 | 1.24 (1.09~1.4) | 0.001 |
| **Severity of disease** |  |  |  |  |
| SAPS II ≥56 | 4.1 (3.79,4.43) | < 0.001 | 1.9 (1.73~2.09) | <0.001 |
| **Organ support therapy** |  |  |  |  |
| RRT | 2.41 (2.15,2.69) | < 0.001 | 1.37 (1.19~1.58) | <0.001 |
| MV | 2.34 (2.13,2.57) | < 0.001 | 2.56 (2.31~2.85) | <0.001 |
| **Clinical data** |  |  |  |  |
| Hemoglobin (g/dL) | 0.96 (0.94,0.97) | < 0.001 | 1.04 (1.02~1.06) | <0.001 |
| WBC (× 10^9^/L) | 1.0084 (1.0068,1.0101) | < 0.001 | 1.01 (1~1.01) | <0.001 |
| Cr (mg/dL) | 1.05 (1.05,1.06) | < 0.001 | 1 (0.98~1.03) | 0.947 |
| BUN (mg/dL) | 1.01 (1.01,1.01) | < 0.001 | 1.01 (1~1.01) | <0.001 |
| PT (s) | 1.02 (1.01,1.02) | < 0.001 | 1 (1~1.01) | 0.089 |
| INR | 1.15 (1.14,1.17) | < 0.001 | 1.03 (0.99~1.08) | 0.171 |
| PTT (s) | 1.0091 (1.0081,1.01) | < 0.001 | 1.01 (1~1.01) | <0.001 |
| HR (bpm) | 1.02 (1.02,1.02) | < 0.001 | 1.02 (1.01~1.02) | <0.001 |
| SBP (mmHg) | 0.98 (0.98,0.98) | < 0.001 | 1 (0.99~1) | 0.065 |
| DBP (mmHg) | 0.991 (0.9871,0.9948) | < 0.001 | 1.01 (1~1.02) | 0.008 |
| MAP (mmHg) | 0.98 (0.98,0.98) | < 0.001 | 0.98 (0.97~1) | 0.008 |
| **Length of stay** |  |  |  |  |
| Length of ICU stay (d) | 1.0067 (1.0025,1.0109) | 0.002 | 1.13 (1.12~1.15) | <0.001 |
| Length of hospital stay (d) | 0.96 (0.95,0.96) | < 0.001 | 0.84 (0.83~0.85) | <0.001 |

Notes: data presented are HRs and 95% CIs.

HRs were adjusted for Age, Sex, Ethnicity, and BMI, CHF, COPD, Mliver, Renal, Diabetes, Metastatic tumor, AIDS, Charlson, Abdomen, Bloodstream, Respiratory tract, Urinary tract, Catheter, MV, RRT, SAPS II, Length of ICU stay, Length of hospital stay, Hemoglobin, WBC, Cr, BUN, PT, INR, PTT, HR, SBP, DBP, MAP.

Abbreviations: BMI, body mass index; CHF, congestive heart failure; COPD, chronic obstructive pulmonary disease; Charlson, Modified Charlson comorbidity index; SAPS II, Simplified Acute Physiology Score II; MV, mechanical ventilation; RRT, renal replacement therapy; WBC, white blood cell count; Cr, creatinine; BUN, blood urea nitrogen; PT, prothrombin time; INR, international normalized ratio; PTT, partial thromboplastin time; HR, heart rate; SBP, systolic blood pressure; DBP, diastolic blood pressure; MAP, mean arterial pressure.

**Supplementary Table 4. Relationship between platelets after multiple imputation and 28-day mortality from sepsis**

|  | Low | Very low | Intermediate-low | Normal | P |
| --- | --- | --- | --- | --- | --- |
|  | (100 x 10^9^ to 150 x 10^9^/L) | (<50 x 10^9^/L) | (50 x 10^9^ to 100 x 10^9^/L) | (≥150 x 10^9^/L) |  |
| **28-day mortality** |  |  |  |  |  |
| Number of deaths/total | 942/7881 | 549/1874 | 749/4448 | 2439/17699 |  |
| Crude Model | 1.0 | 2.75 (2.47~3.05) | 1.46 (1.32~1.6) | 1.17 (1.08~1.26) | <0.001 |
| Model 1 | 1.0 | 3.17 (2.85~3.52) | 1.5 (1.36~1.65) | 1.18 (1.09~1.27) | <0.001 |
| Model 2 | 1.0 | 2.4 (2.15~2.68) | 1.3 (1.18~1.43) | 1.15 (1.07~1.25) | <0.001 |
| Model 3 | 1.0 | 1.72 (1.54~1.93) | 1.19 (1.08~1.31) | 1.17 (1.08~1.26) | <0.001 |

Notes: data presented are HRs and 95% CIs.

Model 1: adjusted for age, Sex, Ethnicity, and BMI;

Model 2: further adjusted (from Model 1) for CHF, COPD, Mliver, Renal, Diabetes, Metastatic tumor, AIDS, Charlson, Abdomen, Bloodstream, Catheter, Respiratory tract, Urinary tract;

Model 3: further adjusted (from Model 2) for MV, RRT, SAPS II, Length of ICU stay, Length of hospital stay, Hemoglobin, WBC, Cr, BUN, PT, INR, PTT, HR, SBP, DBP, MAP.

Abbreviations: BMI, body mass index; CHF, congestive heart failure; COPD, chronic obstructive pulmonary disease; SAPS II, Simplified Acute Physiology Score II; MV, mechanical ventilation; RRT, renal replacement therapy; WBC, white blood cell count; Cr, creatinine; BUN, blood urea nitrogen; PT, prothrombin time; INR, international normalized ratio; PTT, partial thromboplastin time; HR, heart rate; SBP, systolic blood pressure; DBP, diastolic blood pressure; MAP, mean arterial pressure.

**Supplementary Table 5. Multiple regression analysis of platelets and acute kidney injury in participants with sepsis**

|  | Low | Very low | Intermediate-low | Normal | P |
| --- | --- | --- | --- | --- | --- |
|  | (100 x 10^9^ to 150 x 10^9^/L) | (<50 x 10^9^/L) | (50 x 10^9^ to 100 x 10^9^/L) | (≥150 x 10^9^/L) |  |
| Number of deaths/total | 3378/4423 | 735/884 | 1902/2396 | 6843/8698 |  |
| Crude Model | 1.0 | 1.53 (1.41~1.65) | 1.14 (1.08~1.21) | 1.09 (1.05~1.14) | 0.004 |
| Model 1 | 1.0 | 1.67 (1.54~1.81) | 1.17 (1.11~1.24) | 1.1 (1.06~1.15) | 0.003 |
| Model 2 | 1.0 | 1.49 (1.38~1.62) | 1.11 (1.05~1.18) | 1.08 (1.03~1.12) | 0.019 |
| Model 3 | 1.0 | 1.33 (1.22~1.45) | 1.07 (1.01~1.13) | 1.09 (1.05~1.14) | 0.001 |

Notes: data presented are HRs and 95% CIs.

Model 1: adjusted for age, Sex, Ethnicity, and BMI;

Model 2: further adjusted (from Model 1) for CHF, COPD, Mliver, Renal, Diabetes, Metastatic tumor, AIDS, Charlson, Abdomen, Bloodstream, Catheter, Respiratory tract, Urinary tract;

Model 3: further adjusted (from Model 2) for MV, RRT, SAPS II, Length of ICU stay, Length of hospital stay, Hemoglobin, WBC, Cr, BUN, PT, INR, PTT, HR, SBP, DBP, MAP.

Abbreviations: BMI, body mass index; CHF, congestive heart failure; COPD, chronic obstructive pulmonary disease; SAPS II, Simplified Acute Physiology Score II; MV, mechanical ventilation; RRT, renal replacement therapy; WBC, white blood cell count; Cr, creatinine; BUN, blood urea nitrogen; PT, prothrombin time; INR, international normalized ratio; PTT, partial thromboplastin time; HR, heart rate; SBP, systolic blood pressure; DBP, diastolic blood pressure; MAP, mean arterial pressure.

**Supplementary Table 6. Multiple regression analysis of platelets and disseminated intravascular coagulation in participants with sepsis**

|  | Low | Very low | Intermediate-low | Normal | P |
| --- | --- | --- | --- | --- | --- |
|  | (100 x 10^9^ to 150 x 10^9^/L) | (<50 x 10^9^/L) | (50 x 10^9^ to 100 x 10^9^/L) | (≥150 x 10^9^/L) |  |
| Number of deaths/total | 26/4423 | 116/884 | 96/2396 | 43/8698 |  |
| Crude Model | 1.0 | 28.26 (18.46~43.25) | 7.25 (4.7~11.18) | 0.86 (0.53~1.41) | <0.001 |
| Model 1 | 1.0 | 25.41 (16.52~39.08) | 7.05 (4.57~10.88) | 0.8 (0.49~1.3) | <0.001 |
| Model 2 | 1.0 | 19.14 (12.25~29.91) | 6.08 (3.92~9.43) | 0.81 (0.5~1.33) | <0.001 |
| Model 3 | 1.0 | 13.42 (8.48~21.23) | 5.73 (3.67~8.93) | 0.89 (0.54~1.45) | 0.007 |

Notes: data presented are HRs and 95% CIs.

Model 1: adjusted for age, Sex, Ethnicity, and BMI;

Model 2: further adjusted (from Model 1) for CHF, COPD, Mliver, Renal, Diabetes, Metastatic tumor, AIDS, Charlson, Abdomen, Bloodstream, Catheter, Respiratory tract, Urinary tract;

Model 3: further adjusted (from Model 2) for MV, RRT, SAPS II, Length of ICU stay, Length of hospital stay, Hemoglobin, WBC, Cr, BUN, PT, INR, PTT, HR, SBP, DBP, MAP.

Abbreviations: BMI, body mass index; CHF, congestive heart failure; COPD, chronic obstructive pulmonary disease; SAPS II, Simplified Acute Physiology Score II; MV, mechanical ventilation; RRT, renal replacement therapy; WBC, white blood cell count; Cr, creatinine; BUN, blood urea nitrogen; PT, prothrombin time; INR, international normalized ratio; PTT, partial thromboplastin time; HR, heart rate; SBP, systolic blood pressure; DBP, diastolic blood pressure; MAP, mean arterial pressure.

**Supplementary Table7.** Stratified analyses of the associations (hazard ratios, 95% CIs) between Platelet Count and 28-day mortality.

| **Subgroup** | Low | Very low | Intermediate-low | Normal | P _trend_ | P_interaction_ |
| --- | --- | --- | --- | --- | --- | --- |
|  | (100x 10^9^ to 150 x 10^9^/L) | (<50x10^9^/L) | (50x10^9^ to100 x 10^9^/L) | (≥150 x 10^9^/L) |  |  |
| **AKI** |  |  |  |  |  | <0.001 |
| No | 1.0 | 5.28 (3.1~9.02) | 1.7 (1.03~2.79) | 2.1 (1.4~3.13) | 0.011 |  |
| Yes | 1.0 | 1.95 (1.66~2.28) | 1.31 (1.15~1.5) | 1.16 (1.05~1.29) | 0.143 |  |
| **DIC** |  |  |  |  |  | 0.076 |
| No | 1.0 | 1.99 (1.69~2.34) | 1.27 (1.11~1.45) | 1.22 (1.1~1.35) | 0.012 |  |
| Yes | 1.0 | 1.22 (0.61~2.43) | 1.13 (0.57~2.25) | 1.62 (0.77~3.41) | 0.344 |  |

Abbreviations: AKI, [Acute kidney injury](https://www.kidney.org/atoz/content/AcuteKidneyInjury); DIC, disseminated intravascular coagulation.

**Supplementary Table 8. Distributions of variables with missing data comparing observed complete case data to results from pooling the datasets.**

|  | Number (%) with missing data | Missing data | Complete case |
| --- | --- | --- | --- |
| Patients, n (%) | 31902 | 15501 | 16401 |
| **Demographics** |  |  |  |
| Age (mean (SD)) | 66.72 (16.09) | 67.47 (16.54) | 66.00 (15.62) |
| Male (%) | 18331 (57.5) | 8465 (54.6) | 6535 (39.8) |
| White (%) | 21565 (67.6) | 10447 (67.4) | 11118 (67.8) |
| BMI (mean (SD)) | 29.22 (7.78) | 30.34 (9.97) | 29.07 (7.42) |
| **Chronic comorbidity, n (%)** |  |  |  |
| CHF | 10340 (32.4) | 5073 (32.7) | 5267 (32.1) |
| COPD | 8886 (27.9) | 4413 (28.5) | 4473 (27.3) |
| Mliver | 4843 (15.2) | 2322 (15.0) | 2521 (15.4) |
| Renal | 8160 (25.6) | 4270 (27.5) | 3890 (23.7) |
| Diabetes | 7945 (24.9) | 3866 (24.9) | 4079 (24.9) |
| Metastatic tumor | 2018 (6.3) | 1216 (7.8) | 802 (4.9) |
| AIDS | 282 (0.9) | 161 (1.0) | 121 (0.7) |
| Charlson (mean (SD)) | 6.06 (2.94) | 6.25 (2.98) | 5.88 (2.89) |
| **Primary source of infection, n (%)** |  |  |  |
| Abdomen | 77 (0.2) | 28 (0.2) | 49 (0.3) |
| Bloodstream | 2518 (7.9) | 1343 (8.7) | 1175 (7.2) |
| Catheter | 57 (0.2) | 14 (0.1) | 43 (0.3) |
| Respiratory tract | 2116 (6.6) | 540 (3.5) | 1576 (9.6) |
| Urinary tract | 2602 (8.2) | 1318 (8.5) | 1284 (7.8) |
| **Severity of disease** |  |  |  |
| SAPS II (mean (SD)) | 39.73 (14.20) | 38.52 (13.75) | 40.88 (14.53) |
| **Organ support therapy, n (%)** |  |  |  |
| RRT | 1979 (6.2) | 909 (5.9) | 1070 (6.5) |
| MV | 15592 (48.9) | 4883 (31.5) | 10709 (65.3) |
| **Clinical data** |  |  |  |
| Hemoglobin (mean (SD)) | 9.74 (2.14) | 9.83 (2.19) | 9.66 (2.09) |
| WBC (mean (SD)) | 15.26 (12.20) | 14.66 (12.88) | 15.82 (11.50) |
| Cr (mean (SD)) | 1.79 (1.89) | 1.85 (1.94) | 1.74 (1.84) |
| BUN (mean (SD)) | 32.30 (25.24) | 32.83 (25.20) | 31.79 (25.27) |
| PT (mean (SD)) | 18.66 (13.03) | 18.61 (12.99) | 18.70 (13.06) |
| INR (mean (SD)) | 1.72 (1.29) | 1.72 (1.29) | 1.72 (1.29) |
| PTT (mean (SD)) | 44.28 (29.64) | 43.21 (28.69) | 45.16 (30.36) |
| HR (mean (SD)) | 86.93 (16.09) | 86.90 (16.13) | 86.96 (16.05) |
| SBP (mean (SD)) | 115.38 (15.69) | 116.32 (16.63) | 114.49 (14.70) |
| DBP (mean (SD)) | 61.45 (10.44) | 62.13 (10.82) | 60.82 (10.03) |
| MAP (mean (SD)) | 76.33 (10.39) | 76.51 (10.97) | 76.16 (9.81) |
| Platelet (mean (SD)) | 163.00 [111.00, 231.00] | 171.00[114.00, 240.00] | 156.00 [108.00, 221.00] |
